# Surrey Communication and Language in Education Study (SCALES): cohort profile

**DOI:** 10.1136/bmjopen-2025-100710

**Published:** 2025-07-18

**Authors:** Courtenay Norbury, Sarah Griffiths, Laura Lucas, Debbie Gooch, Gillian Baird, Tony Charman, Andrew Pickles, George Vamvakas, Emily Simonoff

**Affiliations:** 1 Psychology and Language Sciences, University College London, London, UK; 2 Special Needs Education, University of Oslo, Oslo, Norway; 3 University of Surrey, Guildford, UK; 4 Newcomen Centre, London, UK; 5 King’s College London, Institute of Psychiatry, London, UK; 6 King’s College London School of Medical Education, London, UK; 7 Department of Biostatistics, King’s College London, London, UK; 8 Child & Adolescent Psychiatry, King’s College London, Institute of Psychiatry, London, UK

**Keywords:** Cognition, Community child health, Developmental neurology & neurodisability

## Abstract

**Abstract:**

**Purpose:**

The Surrey Communication and Language in Education Study (SCALES) cohort was established to estimate prevalence, persistence and impact of developmental language disorders on cognition and mental health, using newly established international consensus diagnostic criteria.

**Participants:**

A population sample of 7267 children aged 4–5 years (59% of eligible children), who started state-maintained school in Surrey, England in 2011–2012 for whom teacher-rated screening data on language, behaviour and early learning goals were available. A subsample of monolingual children enriched for language difficulties completed intensive assessments in year 1 (age 5–6, n=529), year 3 (age 7–8, n=499), year 6 (age 10–11, n=384) and year 8 (age 12–13, n=246). Screening data for 7013 children has been linked to the UK Department of Education National Pupil Database data on special educational needs provision and academic progress.

**Findings to date:**

Language disorders are more prevalent than other neurodevelopmental conditions (such as autism) and more common in areas of socioeconomic disadvantage. Language is a highly stable trait. Language status at school entry is therefore strongly predictive of long-term education progress, the need for specialist support, general cognitive abilities and increased risk for poor mental health, through its effects on social and emotional development.

**Future plans:**

The SCALES cohort will leave compulsory education in 2025 and we are planning to track academic qualifications and post-18 destinations. SCALES data are publicly available via the UK Data Service: DOI: 10.5255/UKDA-SN-8967-1 and DOI: 10.5255/UKDA-SN-8968-1. National Pupil Database data are restricted and cannot be shared. Requests for collaboration and any data that are not publicly available should be addressed to CN, UCL, London (email: c.norbury@ucl.ac.uk).

**STRENGTHS AND LIMITATIONS OF THIS STUDY**

- First UK population study of developmental language disorders using CATALISE consensus diagnostic criteria in a large, inclusive community sample.
- Surrey is a relatively affluent county and thus the numbers of children living in areas of socioeconomic disadvantage are small, limiting the generalisability of our findings.
- There is bias in that parent questionnaires were less likely to be returned by families living in disadvantaged neighbourhoods and/or parenting children with severe language disorder.
- Rates of attrition were impacted by COVID-19 school closures, reducing power to answer questions about predictors of adolescent mental health.

## Introduction

 There is a paucity of research on developmental language disorder (DLD) relative to other neurodevelopmental conditions, despite long-term impacts on health, employment and independence.^1 2^ We established the Surrey Communication and Language in Education Study (SCALES) to provide the first evidence of how internationally agreed diagnostic criteria affected rates of prevalence and persistence of DLD. We also examined the extent to which language difficulties were associated with co-occurring cognitive and behavioural difficulties and adverse mental health during the transition to adolescence. Our purpose was to better understand the mechanisms that link language development and well-being, and to inform policy-makers and practitioners about the prevalence, persistence and pervasive impacts of language disorder.

Previous population studies have focused almost exclusively on school-aged children with ‘specific language impairment (SLI),’ in which children with lower non-verbal cognitive abilities (standard scores <85) were excluded from diagnosis.^3 4^ High rates of co-occurring developmental concerns have resulted in dissatisfaction with the term SLI.^5^ As a result, an international Delphi exercise, CATALISE, agreed changes to diagnostic criteria and terminology,^6 7^ replacing the term SLI with ‘DLD’ as the preferred label for persistent language deficits that occur in the absence of another biomedical condition. ‘Language Disorder (associated with X)’ was the agreed term for cases of language impairment that occurred alongside other developmental conditions, such as autism or Down syndrome. Unlike SLI, the diagnosis of DLD does not require a discrepancy between verbal and non-verbal abilities, and low non-verbal IQ should not preclude diagnosis.

The Early Language in Victoria Study recruited children in infancy and screened for language delay prior to age two.^8^ This study has been highly informative about contextual factors associated with the emergence of language disorder and the longitudinal relationships between language and a range of developmental outcomes. However, this design yielded high rates of resolution of early language delay^9^ and, as a result, is less informative about the developmental patterns associated with persistent language disorder.

SCALES is the only longitudinal, population study to have employed CATALISE criteria. SCALES has comprehensively tracked language, cognition and socioemotional skills for children with heterogeneous developmental profiles from school entry (age 4–5) to adolescence (age 12–13) via direct assessment, multi-informant questionnaires and linkage to national education data.

The overall aims were to:

1. Track the persistence of language disorder identified using newly adopted diagnostic criteria.
2. Establish the developmental relationship between language and non-verbal cognitive skills, and determine whether inclusion of children with lower non-verbal cognition yields a qualitatively different clinical profile of DLD.
3. Document special education provision and education outcomes.
4. Investigate the longitudinal relationship between language and mental health and identify potential mediators of this relationship (eg, emotion processing).

## Patient and public involvement

Our study, information sheets and consent forms were designed in consultation with education staff and parents. The study has benefited from continued engagement with advisory groups composed of education staff, speech-language therapists, families and organisations supporting children and families affected by DLD. Child participants contributed to the development of an animated summary of study findings relating language to mental health.

## Cohort description

All state-maintained schools with a reception class in Surrey, England were invited to take part in 2011–2012 (n=263 schools, n=12 398 children). Income Deprivation Affecting Children Index scores obtained from home postcodes provided a measure of socioeconomic status reflecting neighbourhood deprivation.^10^ Surrey is a relatively affluent county compared with the national average. Index scores in England range from 1 (most deprived) to 32 844 (mean for England in 2010=16 241), and in this sample ranged from 731 (most deprived) to 32 474 (most affluent) (mean=21 592, SD=7830).

Information sheets and opt-out consent forms were distributed to all children (age 4–5) in reception classrooms (20 opt-out forms returned). Complete screening data were returned from 161 schools (61% of eligible schools) for 7267 children (59% of eligible children). There were no differences between schools taking part in the study and those that opted out with regard to the mean percentages of children receiving free school meals, (10.02% vs 8.79%), t(261)=1.38, p=0.17; identified as having special educational needs, (4.89% vs 4.88%), t(261)=0.19, p=0.85; or speaking English as an additional language (EAL), (11.61% vs 10.16%), t(232)=1.05, p=0.29.^11^

Initial stratification identified children who were reported as having ‘no phrase speech’ (NPS) (ie, reported expressive language of two-word utterances or less) (n=89), those attending special schools (n=31) and those for whom EAL (n=777). Children already in special schools were excluded from further study, due to profound learning and often physical needs that teachers felt would make it difficult for them to access our assessments and activities. Children learning EAL were invited to a different study.^12^ All remaining children with NPS (n=48) were invited for in-depth assessment.

For the remaining monolingual children (n=6411), cut-off scores on the Children’s Communication Checklist-Short (CCC-S) (based on^13^) were derived separately for each of three age groups based on season of birth (autumn, spring and summer) to identify strata of boys and girls with teacher ratings of low language (defined as scores at or above the 86th centile for sex and age group). In total, 636 monolingual children (including 48 with NPS) were randomly selected across these strata from state-maintained schools. They were invited to participate with a higher sampling fraction for high-risk (40.5% for boys and 37.5% for girls) vs low-risk (4.3% for boys and 4.2% for girls) children. In year 1 (age 5–6), 529 monolingual children (83% of invited cohort) and families consented to participate and were assessed at school by a member of the research team. Table 1 provides baseline characteristics of those who did and did not respond to invitation into the cohort.

**Table 1:** Comparison of baseline characteristics of children invited into the cohort that were and were not assessed in year 1, and those that responded in year 1 who did and did not meet criteria for language disorder

	Responders			Non-responders
	Typical language	Language disorder	Total	Total
Year 1 (age 5–6)				
n (%)	392	137	529	107
Males n (%)	194 (49)	85 (62)	279 (53)	47 (44)
White n (%)	359 (92)	118 (86)	477 (90)	86 (80)
IDACI mean (SD)	22 428 (7485)	18 320 (7738)	21 364 (7756)	22 012 (7929)
SDQ mean (SD)	7.47 (6.14)	11.76 (6.75)	8.58 (6.57)	11.77 (8.32)
CCC mean (SD)	14.17 (10.3)	27.20 (9.13)	17.55 (11.52)	18.19 (11.27)

CCC, Children’s Communication Checklist; IDACI, Income Deprivation Affecting Children Index; SDQ, Strengths and Difficulties Questionnaire.

**Figure 1:**
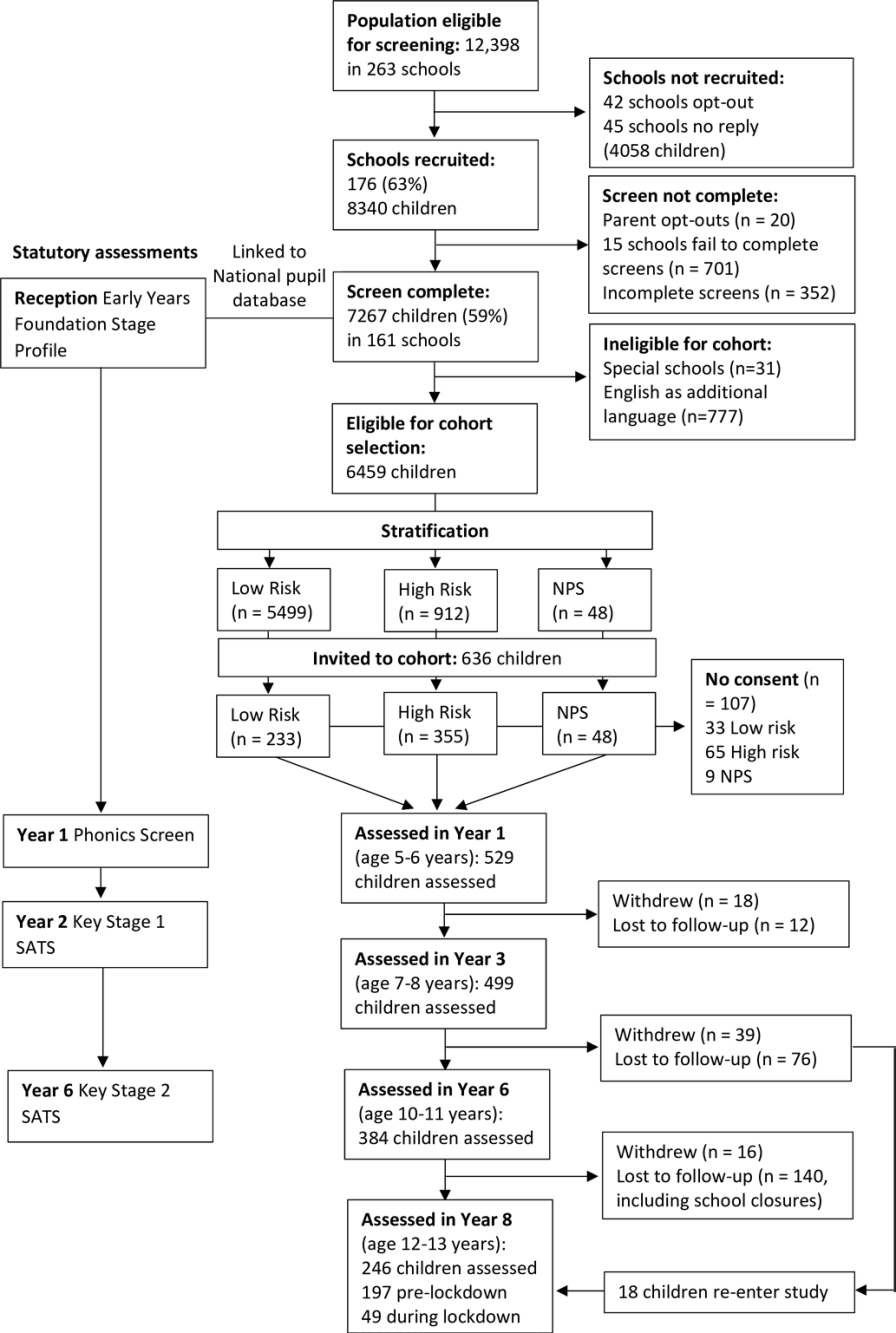
SCALES participant flow chart. NPS, no phrase speech; SATs, Standard Assessment Tests; SCALES, Surrey Communication and Language in Education Study.

There are five waves of in-depth data collection: the initial screening phase (ages 4–5) and four assessments of the intensive cohort (year 1 (age 5–6), year 3 (age 7–8), year 6 (age 10–11), year 8 (age 12–13)). We linked screening data (n=7013) to the National Pupil Database to obtain special educational needs registration and provision from year 1 to year 8, and statutory education assessments in reception, years 1, 2 and 6.^14^ Data collection included direct child assessment, questionnaire responses from children, parents and teachers, and nationally administered tests of academic achievement. Figure 1 illustrates the data collection waves and participant numbers.

### Attrition

General Data Protection Regulation legislation was introduced prior to testing wave 4 and coincided with many children moving from infant to junior schools. This resulted in delays to education authority assistance in tracking children who had moved schools, causing a greater rate of attrition between waves 3 and 4 than between waves 2 and 3. The final testing phase (year 8, age 12–13) was adversely affected by COVID-19 and mandatory school closures. Table 2 lists participant characteristics for those with complete and missing data for both direct assessment and parent/teacher questionnaires at each testing wave.

**Table 2:** Comparison of baseline characteristics of children who left the study in years 3, 6 and 8, grouped by year 1 language disorder status

	Typical language		Language disorder	
	Responders	Non-responders	Responders	Non-responders
Year 3 (age 7–8)				
n (%)	370 (94.39)	22 (5.61)	129 (94.16)	8 (5.84)
Males n (%)	179 (48)	15 (68)	80 (62)	5 (63)
White n (%)	338 (91)	21 (95)	110 (85)	8 (100)
IDACI mean (SD)	22 248 (7435)	25 461 (7847)	18 415 (7780)	16 779 (7321)
SDQ mean (SD)	7.44 (6.12)	8.05 (6.51)	11.60 (6.77)	14.25 (6.30)
CCC mean (SD)	14.08 (10.27)	15.82 (11.01)	26.91 (9.14)	31.88 (8.03)
Year 6 (age 10–11)				
n (%)	281 (75.54)	91 (24.46)	103 (78.63)	28 (21.37)
Males n (%)	134 (48)	47 (52)	62 (60)	20 (71)
White n (%)	259 (92)	79 (87)	90 (87)	20 (71)
IDACI mean (SD)	22 435 (7548)	21 670 (7160)	18 703 (7616)	17 624 (8259)
SDQ mean (SD)	7.07 (5.77)	8.76 (7.1)	12.13 (7.05)	10.04 (5.30)
CCC mean (SD)	13.83 (10.77)	14.86 (8.42)	27.36 (9.33)	25.50 (8.44)
Year 8 (age 12–13)				
n (%)	172 (61.21)	109 (38.79)	53 (51.46)	50 (48.54)
Males n (%)	82 (47)	52 (48)	31 (58)	31 (62)
White n (%)	162 (94)	97 (89)	49 (92)	41 (82)
IDACI mean (SD)	22 803 (7341)	21 854 (7862)	20 030 (7293)	17 296 (7769)
SDQ mean (SD)	6.97 (5.88)	7.23 (5.62)	12.83 (7.48)	11.38 (6.56)
CCC mean (SD)	12.81 (10.27)	15.44 (11.37)	29.08 (9.25)	25.54 (9.16)

CCC, Children’s Communication Checklist; IDACI, Income Deprivation Affecting Children Index; SDQ, Strengths and Difficulties Questionnaire.

### Methods and data collected

#### Core language battery

Language abilities were assessed using a battery of six core language tests in years 1 and 3:^9^ Receptive and Expressive One Word Picture Vocabulary Tests;^15^ a condensed 40-item version of the Test for Reception of Grammar^16^ and the School-Aged Sentence Imitation Test,^17^ a measure of expressive grammar; narrative recall and comprehension using the Assessment of Comprehension and Expression (ACE) (ACE 6–11).^18^

DLD was defined as obtaining scores of −1.5 SD or more on 2 of 5 language composite scores (Vocabulary, Grammar, Narrative, Expressive total, Receptive total) in the absence of intellectual disability (non-verbal cognition composite of <−2 SD) or an existing diagnosis. Language Disorder associated with another biomedical condition was defined as meeting the same language criteria in the context of non-verbal cognition <−2 SD and/or existing diagnosis (eg, autism, known genetic conditions).

#### Child characteristics

Teachers and parents completed questions about the child’s language development, diagnosis and intervention receipt. Parents reported family history of language, literacy and/or learning difficulties and parental levels of education and employment. Children’s communication behaviours were assessed using the CCC-2 (Bishop, 2003) by teachers in year 1 and year 8 and parents at year 1. The CCC-Short^19^ was completed by teachers in reception, year 3 and year 6, and parents in year 3 and year 6. The complete list of available data at each testing wave is listed in table 3.

**Table 3:** Summary of domains measured at each time point of data collection

Child measures	Source	T1	T2	T3	T4	T5
Core language						
Receptive and expressive vocabulary (ROWPVT, EOWPVT)	CA		●	●	●	●
Narrative recall and comprehension (ACE-R, ACE-C)	CA		●	●	●	
Sentence repetition (SASIT)	CA		●	●	●	
Grammar (TROG)	CA		●	●		
Other measures						
Children’s Communication Checklist (CCC-2/CCC-Short)	TQ, PQ	●	●	●	●	●
Non-verbal reasoning	CA		●	●	●	●
Cognitive ability (eg, Attention/Executive Function/speed of processing)	CA		●	●	●	●
Early literacy skills (eg, Letter Sound Knowledge, Phonological awareness, rapid automatised naming)	CA		●	●		
Literacy (word/non-word reading)	CA		●	●	●	●
Literacy (Reading comprehension)	CA			●		●
Social, Emotional, Behaviour Difficulties	TQ,PQ,CQ	●	●	●	●	●
Statutory Educational Attainment (Early Years Foundation Stage Profile, Phonics Screen, Standard Assessment Tests)	NPD	●	●	●		●
Family history	PQ		●	●	●	●
Diagnosis and intervention receipt	PQ,TQ		●	●	●	●
Mental Health & well-being	CQ, PQ				●	●
Emotion processing	CA, CQ				●	●
School life satisfaction	CQ,TQ		●	●	●	●

ACE-R/C, recall and comprehension using the Assessment of Comprehension and Expression; CA, Child Assessment; CQ, Child Questionnaire; NPD, National Pupil Database; PQ, Parent Questionnaire; ROWPVT/ EOWPVT, Receptive and Expressive One Word Picture Vocabulary Tests; SASIT, School-Aged Sentence Imitation Test; TQ, Teacher Questionnaire; TROG, Test for Reception of Grammar.

#### Cognitive ability

At each assessment, children completed Block Design and Matrix Reasoning subtests of the WPPSI-III (year 1)^20^ or WISC-V (years 3, 6, 8)^21^ to assess non-verbal reasoning skills. Experimental measures of executive function assessed verbal and visual working memory, sustained attention, inhibition, task switching and speed of processing.^22^

#### Educational attainment

At reception (age 4–5), teachers completed the Early Years Foundation Stage Profile to summarise progress (rated as emerging, expected, exceeding) towards 17 Early Learning Goals, 12 of which were combined to form a government mandated ‘good level of development’. Further statutory assessment data were obtained by linking SCALES screening data to the National Pupil Database: Year 1 National Phonics Screen, Year 2 Key Stage 1 Standard Assessment Tests (SATs) and Year 6 Key Stage 2 SATs. SATs includes assessment of Reading, Writing and Mathematics.

#### Literacy measures

Letter-sound knowledge was assessed in years 1 and 3 and regular word, exception word and non-word reading^23^ was assessed at all time points. An adapted version of the York Assessment for Reading Comprehension^24^ was administered in years 3 and 8.^22^

#### Child behaviour and mental health

Child behaviour was reported by parents (years 1 and 3), teachers (reception and years 3, 6, 8) and child self-report (years 6 and 8) using the Strengths and Difficulties Questionnaire.^25^ Attention-deficit/hyperactivity disorder symptoms were measured using parent and teacher report in years 1, 3 and 6.^26^ Mental health was reported by parents and children using the Revised Children’s Anxiety and Depression Scale^27^ in years 6 and 8^28^ and the Multidimensional Students Life Satisfaction Scale^29^ in year 8.

#### Emotion recognition and regulation

Children completed experimental tasks of emotion processing in years 6 and/or 8. Emotion regulation was assessed using an experimental temporal distancing task, in which children imagined the impact of an emotional stressor at a later point in time.^30^ Children also completed two tasks of emotion recognition requiring identification of emotions from facial expression or tone of voice cues.^31^ Children also completed questionnaires about emotion regulation (year 6)^32^ and emotion awareness (year 8).^33^

## Findings to date

### Language disorder identified using new consensus diagnostic criteria is both prevalent and persistent

We estimated that 7.58% of children met criteria for DLD; this represents on average two children in every classroom.^19^ A further 2.34% met criteria for language disorder associated with intellectual disability and/or an existing diagnosis of another biomedical condition. Prevalence estimates were associated with socioeconomic status. Predicted probability of language disorder was 2.5 times greater at the 10th centile of IDACI rank (19%) vs the 90th centile (7%) and was greater for boys versus girls, particularly at lowest IDACI quintiles (Boys Q1=26% vs Q5=8%; Girls Q1=13% vs Q5=5%).^34^

Language in this cohort is stable over time, consistent with other longitudinal cohorts.^35^,^37^ Rates of language growth were parallel in groups with and without language disorder. Thus, while language gaps did not narrow or close over time, they did not appear to widen over the course of primary school. While IDACI rank, non-verbal cognition and child sex predicted language scores in year 1, none of these variables predicted rate of language growth over time.^34 38^

### There are mutual developmental relationships between language and non-verbal cognitive skills

In SCALES, 41% of the children who met the criteria for language disorder in year 1 had standardised non-verbal ability scores between 70 and 85, demonstrating that language and non-verbal cognitive difficulties commonly co-occur.^38^ We compared children who met the criteria for language disorder with non-verbal ability scores above and below a standard score of 85. Those with below average non-verbal IQ scores did not differ from those with an ‘SLI’ profile in severity of language deficit or functional impact (eg, behavioural and academic measures). This supports the adoption of the CATALISE^6^ criteria.

Bivariate latent change score models using data from years 3, 6 and 8 found that initial vocabulary scores predicted growth in non-verbal cognition and similarly, initial non-verbal ability scores predicted growth in vocabulary, and these mutualistic relationships were intact in children with language disorder. Thus, excluding children from diagnosis and specialist speech-language support on the basis of non-verbal IQ scores is not warranted.^39^

### Children with language disorder have more specialist education provision, but provision is inconsistent. Children with language disorder have lower levels of academic attainment relative to peers throughout primary school.

Language difficulties at school entry predicted receipt of special educational needs provision, with 68% of children with low language appearing on the Special Educational Needs register during primary school, compared with 19% of peers. Similarly, 8% of children with low language were educated in a specialist setting during primary school compared with 0.5% of peers. However, across the SCALES cohort, special educational needs provision for children with low language is inconsistent, with 38% of children registered as having speech, language and communication needs losing this registration during the transition to secondary school. In contrast, registrations of autism, specific learning difficulties, mild learning disability and social, emotional and mental health increased at transition.^14^

Language difficulties at school entry predicted academic achievement throughout primary school. Teacher-reported language difficulties at screen were moderately correlated with year 1 phonics, r=−0.42; year 2 reading, r=−0.50, writing r=−0.49 and maths r=−0.46; and year 6 reading r=−0.38, writing r=−0.36 and maths r=−0.40 (14). Children with language disorder as a group performed below expectations in all academic assessments, with only 12% achieving a ‘good level of development’ in the first year of school^19^ and as a group, scoring more than 1 SD below peers on statutory education assessments in years 2 and 6.

### Language disorder is associated with increased risk of poor mental health, in part due to the role of language in social and emotional development

Children with language disorder were almost twice as likely to be rated by teachers as having social, emotional or behavioural problems (SEB) on the SDQ relative to peers in Year 1 (TD 5.2%; DLD 9.7%; LD 51.4%).^19^ Language disorder in year 1 was associated with higher teacher-rated SEB problems in year 3 even after adjustment for prior SEB problems, indicating that early language challenges may be causally related to later SEB difficulties.^40^

Children with language disorder also had elevated parent-rated symptoms of anxiety and depression in early adolescence compared with their peers.^28^ In contrast, children with language disorder did not report greater symptoms on self-report measures. We found low agreement between child and parent report across the whole cohort, a common issue in studies that have measured adolescent mental health through both self and parent report.^41^

Early language difficulties were associated with poorer emotion recognition^31^ and emotion regulation^30^ skills in year 6. Similarly, children with language disorder reported having fewer positive and negative social experiences in year 6, indicative of social isolation.^28^ Taken together, these findings suggest a hypothesis that early language difficulties may lead to increased mental health issues in adolescence, partly through the effect of early language on the development of social and emotional skills that are important foundations for good mental health.^42^ Future intervention studies that target early language would provide a robust test of this causal hypothesis.

## Collaboration

Most data are publicly available via the UK Data Archive.^43 44^ However, statutory education assessment data obtained via the National Pupil Database are restricted data and are not publicly available. Some information is not yet in the public domain (ie, individual items for questionnaire data; transcripts of narrative tasks). Parties interested in exploring item level or questionnaire data in more detail should contact Professor Courtenay Norbury (email: c.norbury@ucl.ac.uk).

## Strengths and limitations

The strengths of our study are that it is the first to use CATALISE consensus diagnostic criteria for DLD and Language Disorder associated with other biomedical conditions. The study is also enhanced by the large, inclusive community sample with variable non-verbal cognitive abilities, and the inclusion of multiple measures of functional impact, cognition, behaviour and mental health at each time point, combining experimental, standardised assessment and questionnaire data. This allows assessment of dynamic developmental relationships across domains.

Our conclusions are limited by the fact that Surrey is a relatively affluent county and thus the numbers of children living in areas of socioeconomic disadvantage are small, limiting the generalisability of our findings. In addition, there is bias in parent report measures, because parent questionnaires were less likely to be returned by families living in disadvantaged neighbourhoods and/or parenting children with severe language disorders. Rates of attrition were impacted by COVID-19 school closures, reducing power to answer questions about predictors of adolescent mental health. Finally, information about speech-language therapy provision is limited because this service is mandated by health and not documented in the National Pupil Database. We are, therefore, unable to comment on the potential of speech-language interventions to influence developmental trajectories.

## Data Availability

Data are available in a public, open access repository.
