# Supplementary material for: Surrey Communication and Language in Education Study (SCALES): cohort profile
Source: BMJ Open. 2025 Jul 18;15(7):e100710. doi: 10.1136/bmjopen-2025-100710 (PMC12278150; doi:10.1136/bmjopen-2025-100710)
Supplement: Supplementary file 1 [file bmjopen-2025-100710.reviewer_comments.pdf]

PEER REVIEW HISTORY

BMJ Open publishes all reviews undertaken for accepted manuscripts. Reviewers are asked to complete a checklist review form and are provided with free text boxes to elaborate on their assessment. These free text comments are reproduced below.

ARTICLE DETAILS

Title (Provisional)

Cohort Profile: The Surrey Communication and Language in Education Study (SCALES)

Authors

Norbury, Courtenay; Griffiths, Sarah; Lucas, Laura; Gooch, Debbie; Baird, Gillian; Charman, Tony; Pickles, Andrew; Vamvakas, George; Simonoff, Emily

---

VERSION 1 - REVIEW

**Reviewer**	**1**
**Name**	**Lu, Hsin-Hui**
**Affiliation**	**Chang Gung University**
**Date**	**15-Mar-2025**
**COI**	**None**

This study focuses on the cognitive and mental health development of children with developmental language disorder (DLD) in the future. We utilize the SCALES cohort to investigate this issue. The SCALES cohort is a large-sample longitudinal project, and at different time points, it selects data reported by parents or teachers based on the reliability of the source of children's developmental information. Therefore, analyzing this database can enhance the reliability and validity of the research findings. I have the following questions:

1. In the paper, "language disorders" is commonly used, but "developmental language disorders" appears in four places (Abstract, STRENGTHS AND LIMITATIONS, the first line of Introduction, PATIENT AND PUBLIC INVOLVEMENT). Was there a specific reason for not consistently using "developmental language disorders" in these instances?
2. Although "language disorders" is frequently used, "language disorder" appears twice (Abstract and the second paragraph of 'There are mutual developmental relationships between language and non-verbal cognitive skills'). What was the rationale for not consistently using "language disorders" in these instances?
3. "The overall aims were to:" None of the aims listed have a period at the end. Additionally, could you provide further details on the hypotheses to be tested for these aims?
4. Measures of cognitive ability and child behavior and mental health should use standardized tests and questionnaires. Please report the reliability and validity of these

tests and questionnaires.

5. What is the response format for the emotion recognition and regulation task? Were these tasks developed specifically for the longitudinal study, or do they use other published tasks? Are there any reliability or validity indicators for these tests, such as internal consistency?
6. The "Methods and Data Collected" section only describes what tests and questionnaires measure which indicators at each time point but does not explain the data analysis methods.
7. In the phrase "……Phonics, r=-0.42 [-0.40, -0.44];……", what does "[-0.40, -0.44]" indicate?
8. The paper lacks a complete "Discussion" section to discuss the research findings, including the implications of the main findings, future research directions, and policy implications. It is also unclear what the main limitations of the study are.
9. In Tables 1 and 2, the group sizes "N" should be changed to "n."
10. Table 2 lists the number and performance of participants who continued in the follow-up and were included in the analysis, and those missing, across different demographic and main analysis variables. Has there been further analysis to determine if there are no differences between these two groups on these variables?

**Reviewer**	**2**
**Name**	**Hansson, Kristina**
**Affiliation**	**Lund University, Dept of Clinical Sciences, Lund**
**Date**	**24-Apr-2025**
**COI**	**None**

The profile describes the creation and characteristics of a British cohort of children, followed from school entry at age 4-5 to age 12-13, with a further planned follow-up at age 18. So far, data have been collected at five time points. After screening of 7267 children 636 children were invited to the cohort for extensive assessments and 246 children completed the assessment at age 12-13. The aim is to investigate the prevalence of developmental language disorders, using current diagnostic criteria; how persistent they are and how they impact aspects of cognition and mental health. The main results to date are summarized. Prevalence is found to be equivalent to results from earlier studies (around 7%). The results also show that the condition is persistent. The findings indicate that the current diagnostic criteria applied are appropriate. Furthermore, it is shown that language and non-verbal cognitive skills are mutually interdependent and that children with the disorder are less successful than peers in school and are more at risk of poor mental health.

The project is ambitious and provides/will provide important information to practitioners, researchers and, importantly, policymakers.

I find that the abstract provides the information expected. The Introduction describes the rationale for the project and how it fills a gap in relation to earlier population studies, thus providing motivation for the project. The description of the creation of the cohort is

detailed, and not always easy to follow, since the process, consisting of several steps, was complex, but Figure 1 helps. Data consist of direct assessments of language, literacy and cognitive skills, questionnaires for parents, teachers and the children and data are also retrieved from the National Pupil Database. The battery of tests and the questionnaires are carefully selected and relevant for the purpose of the project, as is the procedure in its entirety. The overview in table 3 is very informative with respect to what data were collected at which time point. The main results are summarized in a way that makes them clearly stand out. The strengths and limitations reported are well chosen. The majority are on the limitations side, and concern things that are difficult to control for.

Some line-by-line comments:

p. 5, line 2 from bottom: I wonder if 731 should be 7,31?

p. 6, first line of 2nd paragraph: if I understand it correctly, NPS (e.g. in the next paragraph) stands for 'no phrase speech', which could be explained here.

p. 6, 2nd paragraph: it is easy to understand that children with EAL were invited to a different study and not included here, but exclusion of children in special schools could need an explanation.

p. 6, 2nd line of 3rd paragraph: other references are given as numbers, so why not (13) here?

p. 6, 3rd paragraph, lines 6-7: 636 children out of 6459 were invited to participate, were they selected by chance, or was there any principle behind who was invited?

p. 8, line 3 from bottom: I don't think SATS has been written out.

This is a very interesting project which can provide much-needed information on the group of children with developmental language disorder. The project/procedure is complex, but this short description gives a good picture.

VERSION 1 - AUTHOR RESPONSE

Reviewer: 1
Dr. Hsin-Hui Lu, Chang Gung University
Comments to the Author:
This study focuses on the cognitive and mental health development of children with developmental language disorder (DLD) in the future. We utilize the SCALES cohort to investigate this issue. The SCALES cohort is a large-sample longitudinal project, and at different time points, it selects data reported by parents or teachers based on the reliability of the source of children's developmental information. Therefore, analyzing this database can enhance the reliability and validity of the research findings. I have the following questions:
1. In the paper, "language disorders" is commonly used, but "developmental language disorders" appears in four places (Abstract, STRENGTHS AND LIMITATIONS, the first line of Introduction, PATIENT AND PUBLIC INVOLVEMENT). Was there a specific reason for not consistently using

"developmental language disorders" in these instances?
Yes – as noted on page 3, language disorder refers to a broader population in which language is affected (i.e. children with autism frequently have language disorder associated with autism). DLD is used to refer to those children who experience language difficulties in the absence of other conditions. SCALES has included both and so it is necessary at to differentiate

2. Although "language disorders" is frequently used, "language disorder" appears twice (Abstract and the second paragraph of 'There are mutual developmental relationships between language and non-verbal cognitive skills'). What was the rationale for not consistently using "language disorders" in these instances?
They are used interchangeably throughout the manuscript

3. "The overall aims were to:" None of the aims listed have a period at the end. Additionally, could you provide further details on the hypotheses to be tested for these aims?

I have put periods at the end of each aim. I do not believe it makes sense to include hypotheses for these aims as we are largely documenting evidence, estimating prevalence and testing associations between language and particular outcomes. For instance, it isn't clear what a hypothesis for special education provision would be - we know young people with language disorders are more likely to be receiving special help at school but there is no way to predict the type or intensity of that support a priori.

4. Measures of cognitive ability and child behavior and mental health should use standardized tests and questionnaires. Please report the reliability and validity of these tests and questionnaires.

There isn't space within journal word limits to provide that level of detail. All of that information is in our meta-data for data held at the UK Data Archive. I have included this sentence in our data availability statement to make that clear:
Meta-data are available for each release and include detailed information about the measures used, including their reliability.

5. What is the response format for the emotion recognition and regulation task? Were these tasks developed specifically for the longitudinal study, or do they use other published tasks? Are there any reliability or validity indicators for these tests, such as internal consistency?

This is an overview paper. All details about the methods and materials are available in the original manuscript which is cited here.

6. The "Methods and Data Collected" section only describes what tests and questionnaires measure which indicators at each time point but does not explain the data analysis methods.

The data analysis methods will vary according to the tasks and the questions. The point of this paper is to let people know what data we have collected and what is available should they wish to conduct their own analyses with our data.

7. In the phrase "……Phonics, r=-0.42 [-0.40, -0.44];……", what does "[-0.40, -0.44]" indicate?

Confidence intervals. However, as we only report CI for this variable I have removed those to save

confusion.

8. The paper lacks a complete "Discussion" section to discuss the research findings, including the implications of the main findings, future research directions, and policy implications. It is also unclear what the main limitations of the study are.

The guidance for Cohort Profiles does not include a Discussion section as results are discussed in the text. The Limitations are included in the bullet point section and I have now included a paragraph about strengths and limitations and their implications.

9. In Tables 1 and 2, the group sizes "N" should be changed to "n."
Done.

10. Table 2 lists the number and performance of participants who continued in the follow-up and were included in the analysis, and those missing, across different demographic and main analysis variables. Has there been further analysis to determine if there are no differences between these two groups on these variables?
This information is provided in Table 2.

Reviewer: 2
Dr. Kristina Hansson, Lund University
Comments to the Author:
The profile describes the creation and characteristics of a British cohort of children, followed from school entry at age 4-5 to age 12-13, with a further planned follow-up at age 18. So far, data have been collected at five time points. After screening of 7267 children 636 children were invited to the cohort for extensive assessments and 246 children completed the assessment at age 12-13. The aim is to investigate the prevalence of developmental language disorders, using current diagnostic criteria; how persistent they are and how they impact aspects of cognition and mental health. The main results to date are summarized. Prevalence is found to be equivalent to results from earlier studies (around 7%). The results also show that the condition is persistent. The findings indicate that the current diagnostic criteria applied are appropriate. Furthermore, it is shown that language and non-verbal cognitive skills are mutually interdependent and that children with the disorder are less successful than peers in school and are more at risk of poor mental health.

The project is ambitious and provides/will provide important information to practitioners, researchers and, importantly, policymakers.

I find that the abstract provides the information expected. The Introduction describes the rationale for the project and how it fills a gap in relation to earlier population studies, thus providing motivation for the project. The description of the creation of the cohort is detailed, and not always easy to follow, since the process, consisting of several steps, was complex, but Figure 1 helps. Data consist of direct assessments of language, literacy and cognitive skills, questionnaires for parents, teachers and the children and data are also retrieved from the National Pupil Database. The battery of tests and the questionnaires are carefully selected and relevant for the purpose of the project, as is the procedure in its entirety. The overview in table 3 is very informative with respect to what data were collected at which time point. The main results are summarized in a way that makes them

clearly stand out. The strengths and limitations reported are well chosen. The majority are on the limitations side, and concern things that are difficult to control for.

Some line-by-line comments:

p. 5, line 2 from bottom: I wonder if 731 should be 7,31?
No, it is 731 (seven hundred and thirty-one

p. 6, first line of 2nd paragraph: if I understand it correctly, NPS (e.g. in the next paragraph) stands for ‘no phrase speech’, which could be explained here.

I have spelled NPS out to avoid confusion

p. 6, 2nd paragraph: it is easy to understand that children with EAL were invited to a different study and not included here, but exclusion of children in special schools could need an explanation.

I have reworded this sentence as follows to provide an explanation:
Children already in special schools were excluded from further study, due to profound learning and often physical needs that teachers felt would make it difficult for them to access our assessments and activities.

p. 6, 2nd line of 3rd paragraph: other references are given as numbers, so why not (13) here?
That is a typo – thanks for spotting it!

p. 6, 3rd paragraph, lines 6-7: 636 children out of 6459 were invited to participate, were they selected by chance, or was there any principle behind who was invited?
I have added this information to that sentence to clarify:
were randomly selected across these strata

p. 8, line 3 from bottom: I don’t think SATS has been written out.
This has been added to the first mention of SATS (Standard Assessment Tests)

This is a very interesting project which can provide much-needed information on the group of children with developmental language disorder. The project/procedure is complex, but this short description gives a good picture.
Thank YOU!

VERSION 2 - REVIEW

**Reviewer**	**1**
**Name**	**Lu, Hsin-Hui**
**Affiliation**	**Chang Gung University**
**Date**	**10-Jun-2025**
**COI**	

No

**Reviewer**	**2**
**Name**	**Hansson, Kristina**
**Affiliation**	**Lund University, Dept of Clinical Sciences, Lund**
**Date**	**19-Jun-2025**
**COI**	

Dear Editor and Authors,

I find that the Authors have responded appropriately to the questions and suggestions raised by the Editor and Reviewers and have no further comments.

Thank you for giving me the opportunity to review this important work!

Yours sincerely
